# A transformer-based multi-task deep learning model for simultaneous T-stage identification and segmentation of nasopharyngeal carcinoma

**DOI:** 10.3389/fonc.2024.1377366

**Published:** 2024-06-14

**Authors:** Kaifan Yang, Xiuyu Dong, Fan Tang, Feng Ye, Bei Chen, Shujun Liang, Yu Zhang, Yikai Xu

**Affiliations:** ^1^ Department of Medical Imaging Center, Nanfang Hospital, Southern Medical University, Guangzhou, Guangdong, China; ^2^ School of Biomedical Engineering, Southern Medical University, Guangzhou, Guangdong, China; ^3^ Guangdong Provincial Key Laboratory of Medical Image Processing, Southern Medical University, Guangzhou, Guangdong, China; ^4^ Guangdong Province Engineering Laboratory for Medical Imaging and Diagnostic Technology, Southern Medical University, Guangzhou, Guangdong, China; ^5^ Department of Radiation Oncology, Nanfang Hospital, Southern Medical University, Guangzhou, Guangdong, China

**Keywords:** nasopharyngeal carcinoma, deep learning, tumor segmentation, T-stage identification, multi-task

## Abstract

**Background:**

Accurate tumor target contouring and T staging are vital for precision radiation therapy in nasopharyngeal carcinoma (NPC). Identifying T-stage and contouring the Gross tumor volume (GTV) manually is a laborious and highly time-consuming process. Previous deep learning-based studies have mainly been focused on tumor segmentation, and few studies have specifically addressed the tumor staging of NPC.

**Objectives:**

To bridge this gap, we aim to devise a model that can simultaneously identify T-stage and perform accurate segmentation of GTV in NPC.

**Materials and methods:**

We have developed a transformer-based multi-task deep learning model that can perform two tasks simultaneously: delineating the tumor contour and identifying T-stage. Our retrospective study involved contrast-enhanced T1-weighted images (CE-T1WI) of 320 NPC patients (T-stage: T1-T4) collected between 2017 and 2020 at our institution, which were randomly allocated into three cohorts for three-fold cross-validations, and conducted the external validation using an independent test set. We evaluated the predictive performance using the area under the receiver operating characteristic curve (ROC-AUC) and accuracy (ACC), with a 95% confidence interval (CI), and the contouring performance using the Dice similarity coefficient (DSC) and average surface distance (ASD).

**Results:**

Our multi-task model exhibited sound performance in GTV contouring (median DSC: 0.74; ASD: 0.97 mm) and T staging (AUC: 0.85, 95% CI: 0.82–0.87) across 320 patients. In early T category tumors, the model achieved a median DSC of 0.74 and ASD of 0.98 mm, while in advanced T category tumors, it reached a median DSC of 0.74 and ASD of 0.96 mm. The accuracy of automated T staging was 76% (126 of 166) for early stages (T1-T2) and 64% (99 of 154) for advanced stages (T3-T4). Moreover, experimental results show that our multi-task model outperformed the other single-task models.

**Conclusions:**

This study emphasized the potential of multi-task model for simultaneously delineating the tumor contour and identifying T-stage. The multi-task model harnesses the synergy between these interrelated learning tasks, leading to improvements in the performance of both tasks. The performance demonstrates the potential of our work for delineating the tumor contour and identifying T-stage and suggests that it can be a practical tool for supporting clinical precision radiation therapy.

## 1 Introduction

Nasopharyngeal carcinoma (NPC), a frequently occurring malignant tumor in the head and neck region (1), primarily receives intensity-modulated radiation therapy (IMRT) as the standard treatment (2). The close proximity of NPC to critical neural and other organs necessitates precise delineation of the primary gross tumor volume (GTV) to minimize the risk of radiation-induced toxicities (3). Typically, early-stage NPC is managed with radiotherapy alone, whereas advanced local-regionally advanced disease often requires a combined approach of radiotherapy and chemotherapy (1, 4). Significantly, errors in contouring and T-staging adversely affect survival rates in head and neck cancer patients (5). Therefore, accurate tumor target contouring and T staging are vital for precision radiation therapy in NPC.

The complex anatomy of the nasopharynx and skull base complicates the precise identification of tumor invasion areas through surgical resection. NPC staging predominantly relies on physician interpretations of diagnostic images (1). Consequently, GTV contouring and T-staging for NPC are not only laborious but also highly time-consuming, requiring meticulous review of multi-modal or multi-parametric imaging data slice-by-slice, even by experienced physicians. Furthermore, GTV contouring and T-staging are prone to errors, particularly since NPC can infiltrate adjacent skull base and neural structures with often subtle signal variations in MRI scans. Thus, the NPC radiation therapy planning process heavily depends on the expertise of the physicians. Therefore, there is a significant clinical need for automatic segmentation and identification of GTV to aid in clinical decision-making, treatment planning, and ongoing tumor monitoring.

Recent advancements in deep-learning-based automatic methods have facilitated automatic NPC GTV segmentation (3, 6–9) and NPC staging (10–12). Lin et al. (3) pioneered the application of deep learning techniques for automating the contouring of primary tumor volumes in NPC patients using MRI. Chen et al. (6) introduced a multi-modality MRI fusion network to achieve precise segmentation of NPC by leveraging T1, T2, and contrast-enhanced T1 MRI images (CE-T1WI). Tang et al. (7) proposed a dual attention mechanism for feature refinement to enhance NPC segmentation accuracy. Tao et al. (8) devised a sequential method for NPC segmentation in MR images, effectively addressing inherent background dominance issues at both the instance and feature levels. Regarding NPC staging, Yang et al. (10) proposed a weakly supervised deep learning model for NPC T-staging. Wong et al. (11) employed a CNN to automatically discriminate early-stage NPC from benign hyperplasia based solely on a non-contrast-enhanced MRI sequence. Huang et al. (12) proposed a two-stage classification framework for predicting locoregionally advanced NPC (stages III and IVa). In clinical practice, sizes and shapes of tumors are various and heterogeneous, assist the radiologists in identifying the four stages of T staging. This important characteristic of positional information is useful for both tumor segmentation and classification. However, existing deep learning-based studies have predominantly focused on either tumor segmentation or tumor staging of NPC alone, overlooking the inherent correlation between tumor segmentation and classification. Recent studies have highlighted the potential of deep learning models, especially those with refined multi-task features, in managing complex clinical tasks related to diseases like breast cancer (13), COVID-19 (14), gastric cancer (15), and thyroid nodules (16). Consequently, an integrated approach for training GTV contouring and T-staging within a unified network, promoting information sharing between these tasks, holds promise.

Convolutional Neural Network (CNN) are particularly adept at capturing spatial hierarchies in images, which makes them highly effective for image segmentation tasks (3, 6–8). The local connectivity and weight sharing in CNNs allow them to excel in extracting features from images that are spatially correlated, making them suitable for delineating complex structures like tumors in medical images. Additionally, the Transformer architecture, equipped with its Multi-Head Self-Attention (MHSA) mechanism (17), has demonstrated outstanding performance in analyzing long sequence correlations. This ability allows Transformers to learn expressive representations and filter out irrelevant signals, thereby significantly enhancing image analysis capabilities. Given the intricacy and distinctiveness of our task, we aim to develop a multi-task model that leverages the strengths of both architectures using contrast-enhanced T1-weighted images (CE-T1WI) that can simultaneously identify T-stage and perform accurate segmentation of GTV in NPC. Specifically, we employ the 3D U-Net architecture for the GTV segmentation task. For T-stage identification, we design a Transformer-style prediction network that is adept at processing complex sequential information. To connect these two distinct tasks, we introduce a feature-fusion-aware module which is designed to analyze correlations among long sequences and learn expressive representations, improving the aggregation of features between the segmentation and staging tasks.

## 2 Materials and methods

### 2.1 Dataset

This retrospective study received approval from the ethics committee of our institution (No. NFEC-2023-417), and the requirement for informed consent was waived. The research was conducted in strict adherence to the guidelines and regulations set by the ethics committee.

Two independent datasets were used in this study. The inclusion criteria was as follows: (1) a definitive histopathological diagnosis of NPC; (2) absence of distant metastasis prior to treatment; (3) no previous history of radiotherapy; and (4) availability of a CE-T1WI sequence.

The first dataset included 356 cases of nasopharyngeal carcinoma. All patients were imaged using MRI platforms from different manufacturers using dissimilar imaging protocols: Optima MR360, GE Medical Systems, USA (n=280); Signa HDxt, GE Medical Systems, USA (n=43); Ingenia, Philips Medical Systems, USA (N=4); Achieva, Philips Medical Systems, USA (n=5); and Avanto, Siemens, Germany (n=24). The image size is (256 - 672) × (256 - 672) × (16 - 120), with in-plane resolution as 0.34 - 1.09 mm, and slice thickness as 3.5 - 7 mm. We excluded 36 cases because of poor image quality (n = 16) or missing data (n = 20). Finally, 320 cases were retrospectively included as Dataset 1. These images were collected using different scanners with different image sizes and resolutions, thus increasing the diversity of the acquired MR images, and further improving the generalization of models.

The second dataset included 172 cases of nasopharyngeal carcinoma. The image size is 512 × 512 × (40 - 76), with in-plane resolution as 0.4688 - 0.5078 mm, and slice thickness as 3 - 6.7 mm. We excluded 22 cases because of poor image quality (n = 16) or missing data (n = 6). Finally, 150 cases were retrospectively included as Dataset 2.

**Figure 1:**
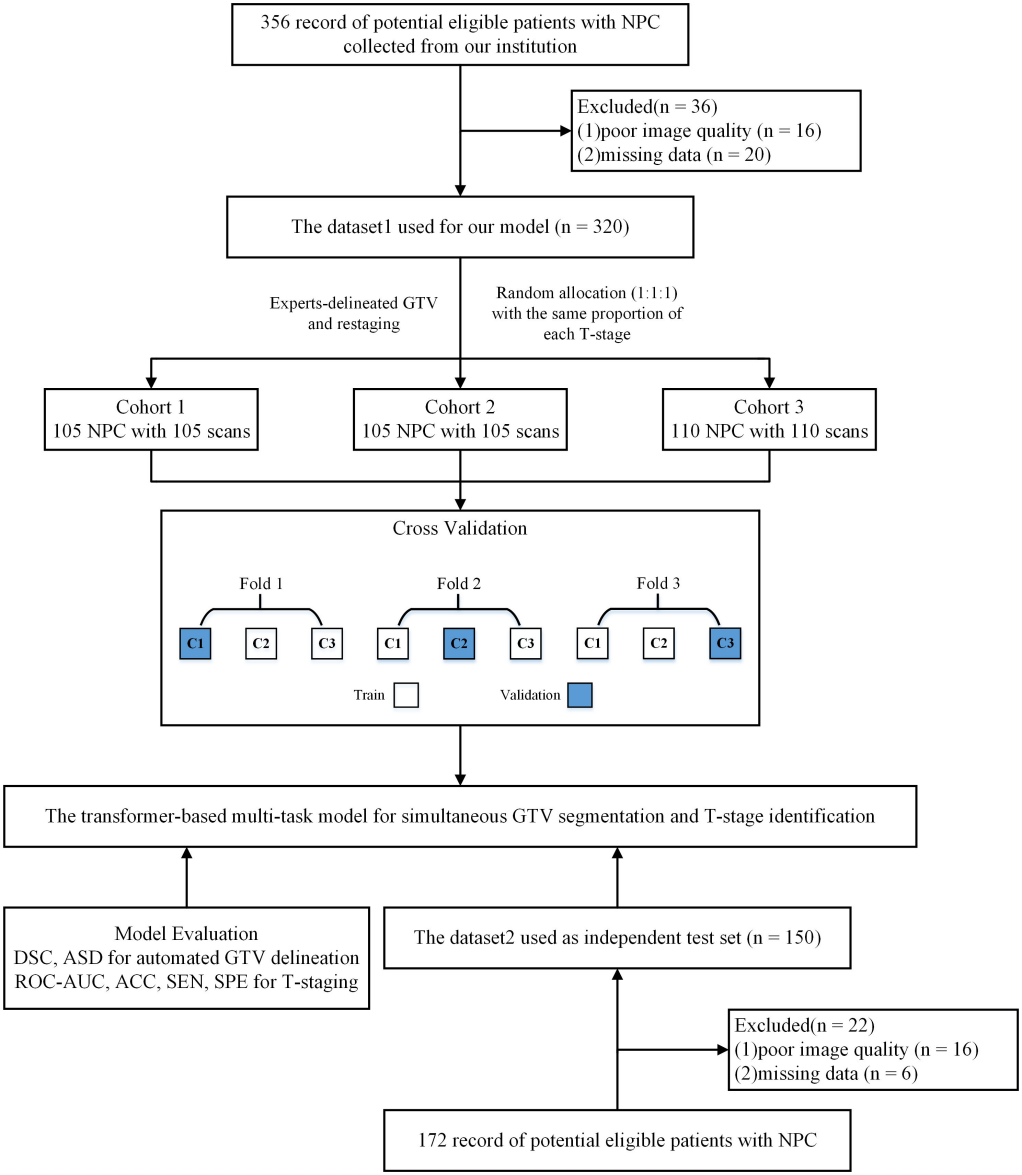
Study flow diagram. NPC, nasopharyngeal carcinoma; DSC, dice similarity coefficient; ASD, average surface distance; ACC, accuracy; SEN, sensitivity; SPE, specificity; ROC-AUC, the area under the receiver operating characteristic curve.

In this study, a three-fold cross-validation strategy was used in Dataset 1 to select the best hyper-parameters. All subjects within Dataset 1 were divided into three subsets with the same proportion of each T-stage. Specifically, in each round, one subset was used for validation and the remaining two subsets were used for training. We repeated this process three times until all three data subsets had served as the validation set. Finally, the best hyper-parameters were obtained, and the models obtained by this three-fold cross-validation strategy were tested on the independent testing set. The research design is illustrated in **Figure 1**.

### 2.2 GTV manual delineation and patient re-staging

Manual segmentation of GTVs on CE-T1WI images were performed by a radiologist with 7 years of experience and checked by a radiation oncologist with more than 10 years of experience. All delineations were performed using the ITK-SNAP software (version 3.8.0, http://www.itksnap.org). These Manual segmentation of GTVs served as the basis for training, validating and testing our multi-task model.

To enhance data consistency and mitigate inter-observer variability, two experienced radiation oncologists (more than 10 years of experience in the field) independently reviewed the MR images and clinical data. Any discrepancies were resolved through a consensus-based approach. The T stages of the patients with NPC were reassessed according to the 8th edition of the Union for International Cancer Control/American Joint Committee on Cancer (UICC/AJCC) staging system (18).

### 2.3 Network architectures

**Figure 2:**
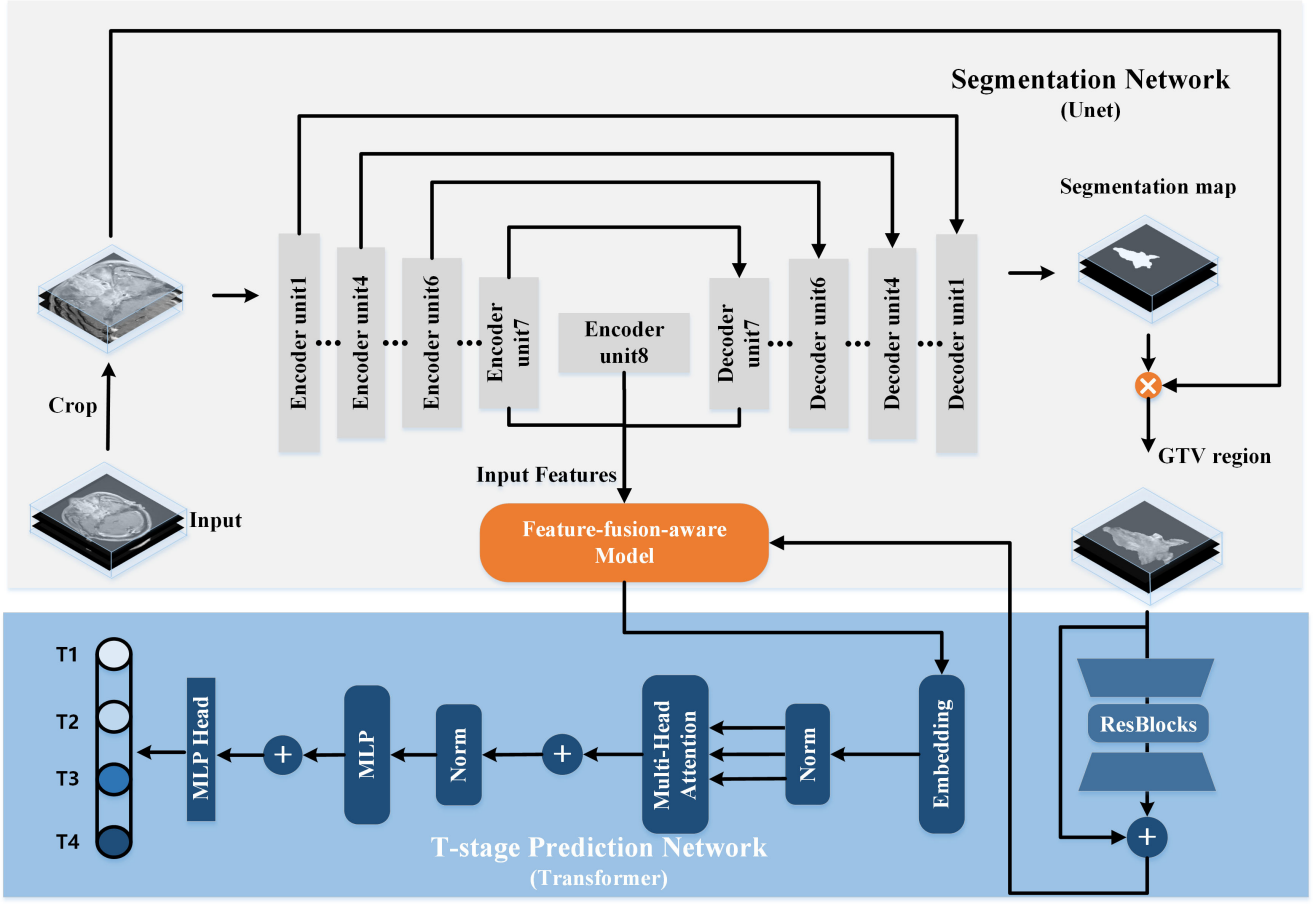
The architecture of our proposed transformer-based multi-task model for simultaneous GTV segmentation and T-stage identification.

We propose a multi-task model designed to simultaneously identify T-stage and accurately segment GTV in NPC. Illustrated in **Figure 2**, the proposed model comprises two primary components: a segmentation network for delineating the regions of interest (ROI) of the GTV, and a T-stage predictor for automated prediction of T-stage probability scores. Within the segmentation network, a modified 3D U-Net is employed to perform GTV segmentation. Additionally, the segmented map is multiplied by the original MR image to generate a GTV region, serving as input for the T-stage predictor. In the T-stage prediction network, the tumor region is taken as input, initiating with a Bottleneck Transformer as the backbone to extract relevant features pertaining to the tumor. Notably, we adapt the network to accommodate and process 3D inputs. Furthermore, a feature-fusion-aware module is devised to connect and synchronize features derived from both GTV segmentation and T-staging processes. Finally, a classifier is employed to predict the probability scores of the four T stages.

#### 2.3.1 3D segmentation network

The architecture of the 3D segmentation network encompasses a U-shaped encoding-decoding network comprising eight encoder units and seven decoder units. Traditionally, lower network layers extract feature maps delineating spatial structure information, while higher network layers furnish rich semantic information. Consequently, we posit that the most representative features learned by the segmentation network reside at the bottom of the U-shaped structure, specifically the seventh and final encoder units and the initial decoder unit. These features are subsequently fed into the T-stage prediction network.

The encoder part constitutes a contracting path facilitating down-sampling of the input image through eight encoder units, each succeeded by a 3D max pooling or average pooling operation with a stride of 2. Conversely, the decoder part embodies an expansive path, where feature maps are up-sampled through seven decoder units employing up-convolution layers. Notably, skip connections play a pivotal role. By establishing connections between feature maps from the compression path to the expansion path, the network assimilates spatial information and refines segmentation predictions.

Subsequently, the output of the last decoder unit undergoes processing by a 1×1×1 convolution followed by a sigmoid function. The generated segmentation map is then multiplied by the original MR image, yielding the input for the T-stage prediction network.

#### 2.3.2 T-stage prediction network

The Transformer architecture, with its Multi-Head Self-Attention (MHSA) mechanism (17), has shown exceptional performance in analyzing long sequence correlations, learning expressive representations, and filtering out irrelevant signals, thus enhancing image analysis capabilities. Motivated by these findings, we design a transformer-style prediction network for T staging of NPC. As illustrated in **Figure 2**, the structure of T-stage prediction network consists of three modules: the 3D bottleneck transformer, the feature-fusion-aware module and a classifier.

*3D Bottleneck Transformer.* We adopt a backbone architecture called Bottleneck Transformer (19), which integrates self-attention mechanisms tailored for our T-stage prediction task. Its conception is simple: replace the final three spatial convolutions in a ResNet (20) with MHSA layers. As we all known, the ResNet structure typically has four stages. Our network just replaces the final three bottleneck blocks of ResNet-50 with MHSA layers.

**Figure 3:**
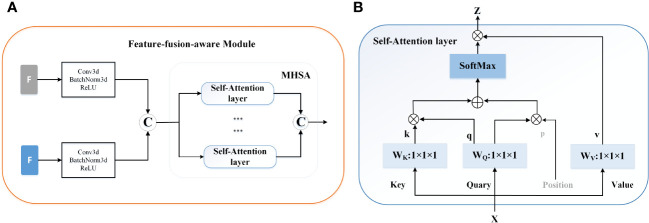
The mechanism of the feature-fusion-aware module. **(A)** The gray ‘F’ represent the features extracted from the segmentation network and the blue ‘F’ indicates the output of the 3D backbone of prediction network. ^©^ represents the concatenation operation. **(B)** The detailed structure of Self-Attention layer.

*Feature-fusion-aware Module.* In **Figure 3A**, our feature-fusion-aware module combines the convolutional layer in CNNs with the MHSA layer in Transformer, which can take into account the features across different tasks. The MHSA layer consists of four parallel self-attention layers, each referred to as a head. For each head, query, key, and value are mapped into three current layers before attention calculation. The output of the four attention heads is then concatenated and input into the last linear layer for integration. In the self-attention layer (**Figure 3B**), the input is mapped to q, k, and v. Subsequently, the spatial position encoding vector is multiplied by q as a spatial prior, and content information is acquired by multiplying q and k. Then, the spatially sensitive similarity feature is obtained by adding the two. Finally, the corresponding attention coefficient of the feature is multiplied by v to yield the final output z. Note that, the symbols ⊕ and ⊗ represent element-wise sum and matrix multiplication respectively, while 1 × 1 × 1 represents a point-wise convolution. And q, k, v and p represent query, key, value and position respectively.

In the final stage of the network, a classifier is deployed to accurately estimate the probability scores for the four distinct T stages.

#### 2.3.3 Loss function

Our proposed method is a multi-task learning model that involves T-staging and tumor segmentation. T-staging can be considered as a classification task. As such, the overall loss function in Equation 1 consists of two components: the classification loss *L_Cls_* for T-staging and the segmentation loss *L_Seg_* for GTV segmentation:


(1) $$L=\lambda_{Seg}L_{Seg}\{p,\overset{⌢}{p}\}+\lambda_{Cls}L_{Cls}\{y,\overset{⌢}{y}\}$$

where *λ_Cls_* and *λ_Seg_* are flexible hyper-parameters updated during network training. In this study, the parameters *λ_Cls_* and *λ_Seg_* are initially set as two tensors with a value of 1, and they are subsequently iteratively updated during the training phase. *L_Seg_* represents a hybrid loss function which is combined by the Jaccard loss and the Focal loss, *p* denotes the ground-truth mask and $\overset{⌢}{p}$ denotes the predicted segmentation map; *L_Cls_* is defined as cross-entropy loss aimed to compare the predicted probability of class with the ground-truth label, *y* denotes the ground-truth label and $\overset{⌢}{y}$ is the prediction probability.

### 2.4 Implementation details

*Image processing:* We resampled the images to a targeted resolution of 0.4688×0.4688×6 mm³, which represents the median spacing of all image data. This resampling utilized third-order spline interpolation for in-plane adjustments and the nearest neighbor approach for out-of-plane interpolation, as per the methodology described in (21). To address potential issues in deep network signal propagation caused by large signals, we normalized each input by subtracting the mean and then dividing by the standard deviation. For the inputs to our multi-task model, we cropped the original MR images to a size of 21×224×224.

*Training detail:* The model was trained on Pytorch, utilizing an NVIDIA RTX 3090 Ti graphics processing unit (GPU), with axial T1CE. The training employed the ‘Adam’ optimizer, which was tasked with optimizing the combined loss. Training parameters included a batch size of four and an initial learning rate of 2 × 10^-4^. To enhance the model’s generalizability and stability, data augmentation techniques (22) and a ReduceLROnPlateau learning rate scheduling strategy (23) were employed. The training process was concluded either when the learning rate fell below 10^-9^ or after completing 300 epochs.

### 2.5 Performance evaluation

The performance of the models was assessed using the Dice similarity coefficient (DSC) and the average surface distance (ASD). The DSC quantifies the spatial overlap between the model-generated contour (A) and the ground truth contour (G), which is defined as $DSC(A,G)=\frac{2\left|A\cap G\right|}{\left|A\right|+\left|G\right|}$ . ASD measures the average distance between the surfaces of two contours. Additionally, The predicted accuracy (ACC), sensitivity (SEN), specificity (SPE) of the model and the radiologists for identifying T-stage were evaluated by calculating the 95% confidence intervals (CIs) using the Clopper-Pearson method. Moreover, the area under the receiver operating characteristic curve (ROC-AUC) was employed to evaluate the algorithm’s capability in predicting the T-stage. This metric is crucial for determining the effectiveness of our multi-task model in classifying the stages of nasopharyngeal carcinoma accurately.

### 2.6 Statistical analyses

For our study, categorical variables across the combined three cross-validation cohorts were compared using the chi-square test. Numeric variables, including comparisons of the DSC and ASD between different subgroups, were analyzed with the Mann-Whitney U test. Furthermore, the Wilcoxon matched-pairs signed rank test was employed for contrasting DSC and ASD values obtained from our model against both manual delineation and other deep learning methods. The DeLong test was utilized for comparing the ROC-AUC metrics, while the McNemar test was applied for assessing the ACC.

Statistical analyses were conducted using the Statistical Product and Service Solutions (IBM SPSS, version 26.0) and open-access Python (version 3.6.10) statistical packages, namely “sklearn”, “scipy”, and “statsmodels”. For all tests, statistical significance was determined based on a two-tailed P< 0.05.

## 3 Results

### 3.1 Patient demographics

Patient demographics of Dataset1 is shown in **Table 1**. The Dataset1 included 320 patients, characterized by a median age of 48 years (range: 19-80 years), comprising 235 men (73.3%) and 85 women (26.6%). Stratification according to the 8th edition of the AJCC/UICC staging system identified 26 (8.1%), 140 (43.8%), 87 (27.2%), and 67 (20.9%) patients with T1-T4 stage disease, respectively. No significant differences in sex, age, and T category were observed between the combined three cross-validation cohorts.

**Table 1:** Characteristics of the study population of Dataset1.

Characteristic	Total (n = 320)	Cohort 1 (n = 105)	Cohort 2 (n = 105)	Cohort 3 (n = 110)	*P* Value		
					C1 vs C2	C1 vs C3	C2 vs C3
Sex, No (%)					0.541	0.407	0.148
Male	235 (73.3)	77 (73.4)	73 (69.5)	85 (77.3)			
Female	85 (26.6)	28 (26.7)	32 (30.5)	25 (22.7)			
Age, median (range), y	48 (19-80)	48 (19-80)	49 (20-74)	48 (19-80)	0.696	0.633	0.925
T stage, No (%)					1	0.962	0.962
T1	26 (8.1)	8 (7.6)	8 (7.6)	10 (9.1)			
T2	140 (43.8)	47 (44.8)	47 (44.8)	46 (41.8)			
T3	87 (27.2)	28 (26.7)	28 (26.7)	31 (28.2)			
T4	67 (20.9)	22 (20.9)	22 (20.9)	23 (20.9)			

Data are either number of patients, with the percentage in parentheses, or median, with the range in parentheses. We calculated P values by using the $\chi^{2}$ test for category variables and the Mann-Whitney U test for numeric variables. Two-tailed P< 0.05 indicated a significant difference. C1, Cohort1; C2, Cohort2; C3, Cohort3.

### 3.2 Model performance in delineating GTV contour

**Figure 4:**
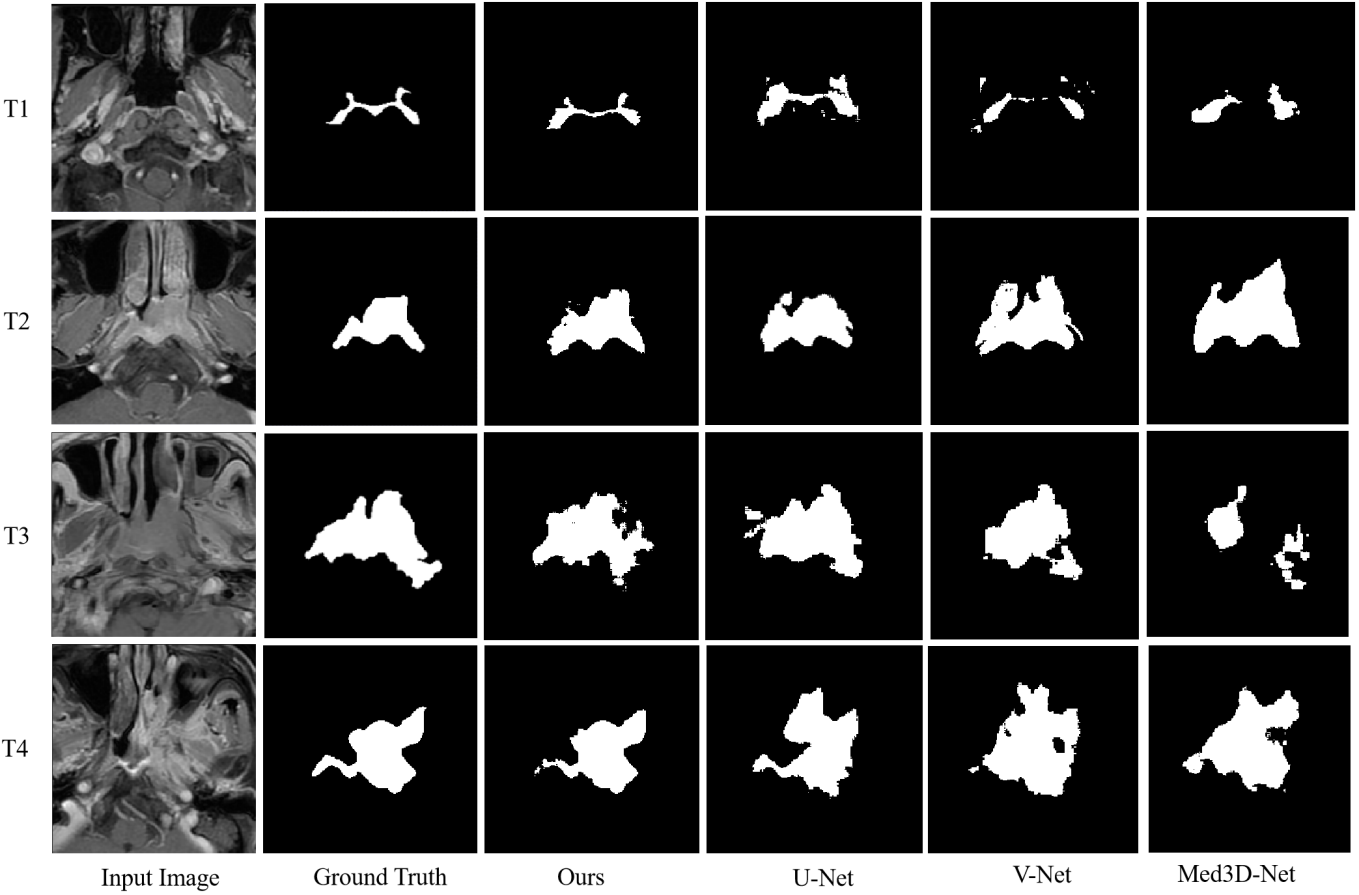
Example contrast-enhanced T1-weighted MRIs show the level of concordance for primary gross tumor volume contours between our proposed multi-task model and 3D U-Net, V-Net, and Med3D Net.

The segmentation performance of our method is summarized in **Table 2**; **Figure 4** illustrates the concordance level for GTV contours between our method and other segmentation methods. The median DSC was 0.74 (IQR, 0.11; 95% CI: 0.73, 0.75), and the median ASD was 0.97 mm (IQR, 0.75 mm; 95% CI: 0.9, 1.02 mm), less than the commonly accepted 3-mm margin of systematic and random error in radiation therapy for head and neck cancers (24). These results indicate strong concordance with human experts in GTV contouring.

**Table 2:** Performance of our multi-task model in delineating GTV contour using Dataset1.

	Total (n = 320)	T Category		*P* Value
		T1 and T2 (n = 166)	T3 and T4 (n = 154)	
DSC				0.648
Median	0.74	0.74	0.74	
IQR	0.11	0.11	0.11	
95% CI	0.73, 0.75	0.73, 0.75	0.72, 0.75	
ASD (mm)				0.984
Median	0.97	0.98	0.96	
IQR	0.75	0.83	0.68	
95% CI	0.90, 1.02	0.87, 1.16	0.89, 1.02	

We calculated the P value by using Mann-Whitney U test. Two-tailed P< 0.05 indicates a significant difference. DSC, Dice similarity coefficient; ASD, average surface distance; IQR, Inter-quartile range; CI, confidence interval.

In different T categories, our multi-task model achieved a median DSC of 0.74 (IQR, 0.11; 95% CI: 0.73, 0.75) and a median ASD of 0.98 mm (IQR, 0.83 mm; 95% CI: 0.87, 1.16 mm) in early T category tumors, and a median DSC of 0.74 (IQR, 0.11; 95% CI: 0.72, 0.75) and a median ASD of 0.96 mm (IQR, 0.68 mm; 95% CI: 0.89, 1.02 mm) in advanced T category tumors. No differences in DSC and ASD were observed across T categories, suggesting robustness of our multi-task model in contouring tumors of various sizes.

We also compared GTV contour accuracy of our multi-task model against other models like 3D U-Net (25), V-Net (26), and Med3D Net (27). As shown in **Table 3**, 3D U-Net showed the best DSC and ASD compared to V-Net, and Med3D Net. However, our multi-task model-generated contours had superior median DSC and ASD compared to those generated with 3D U-Net (median DSC, 0.74 vs 0.73; median ASD, 0.97 vs 1.05 mm; P = 0.013< 0.05 for DSC; **Table 3**).

**Table 3:** Accuracy of contours generated by our proposed multi-task model and the 3D U-Net, V-Net, and Med3D Net.

	DSC			ASD (mm)			*P* Value	
	Median	95% CI	IQR	Median	95% CI	IQR	DSC	ASD
U-Net	0.73	0.72, 0.74	0.12	1.05	0.96, 1.13	0.86	0.013	0.144
V-Net	0.69	0.68, 0.71	0.13	1.26	1.16, 1.36	1.01	< 0.05	< 0.05
Med3D Net	0.65	0.63, 0.67	0.18	1.51	1.42, 1.65	1.55	< 0.05	< 0.05
Ours	0.74	0.73, 0.75	0.11	0.97	0.9, 1.02	0.75		

We calculated P value using Wilcoxon matched-pairs signed-rank test, two-tailed P< 0.05 indicates significant difference. DSC, Dice similarity coefficient; ASD, average surface distance; IQR, inter-quartile range; CI, confidence interval.

### 3.3 Model performance in identifying T-stage

**Figure 5:**
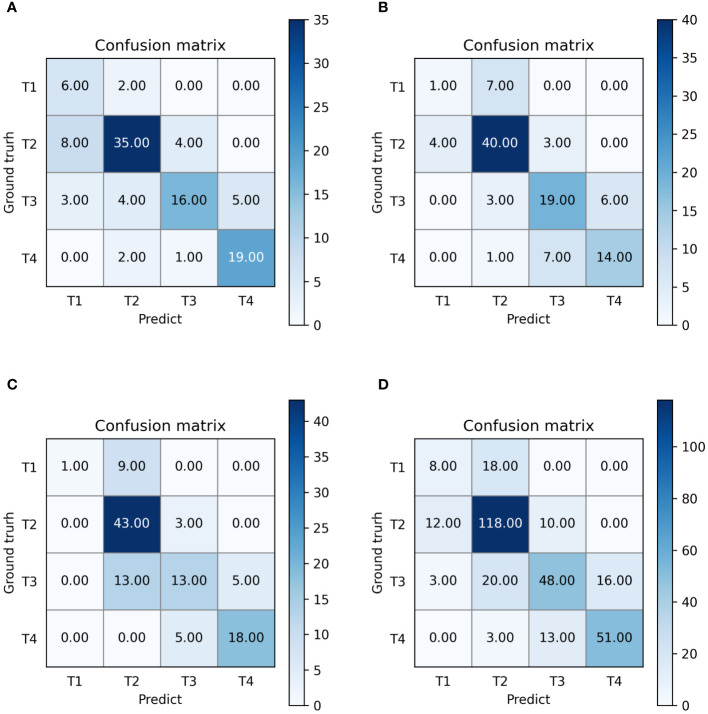
Confusion matrices of our multi-task model. **(A–D)** denote the performance of fold 1-3 and the average result, respectively.

**Figure 6:**
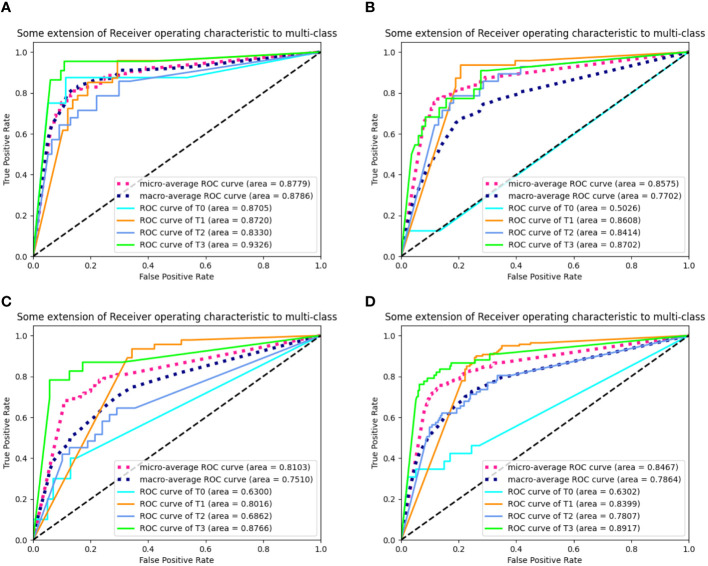
The receiver operating characteristic curves (ROCs) of our model. **(A–D)** denote the performance of fold 1-3 and the average result, respectively. ROC, receiver operating characteristic; AUC, area under curve; Micro-average, calculate metrics globally by considering each element of the label indicator as a label; Macro- average, calculate metrics for each label, and find their un-weighted mean.

The outputs of our multi-task model are the probabilities of stages T1, T2, T3, and T4. Confusion matrices and ROC curves are presented in **Figures 5**, **6**, respectively. We observed an AUC of 0.85 (95% CI: 0.82, 0.87), an ACC of 0.7 (225 of 320; 95% CI: 0.65, 0.75), a SPE of 0.89 (95% CI: 0.88, 0.9), and a SEN of 0.62 (95% CI: 0.61, 0.62) as per **Table 4**. Three-fold cross-validation revealed ACCs of 0.72, 0.7, and 0.68, respectively (**Figure 5**), and AUCs of 0.88, 0.86, and 0.81, respectively (**Figures 6A–C**). For early T1-2 stages, automated T staging accuracy was 0.76 (126 of 166), while for advanced T3-4 stages, it was 0.64 (99 of 154), indicating a significant difference between early and advanced stages (P< 0.05).

**Table 4:** Accuracy of T-staging by our proposed multi-task model and the 3D ResNet-50, RAN, DenseNet and ResNext.

	AUC	ACC	SPE	SEN	P^*^	*P^#^*
ResNet	0.80 (0.77, 0.83)	0.59 (0.54, 0.64)	0.85 (0.84, 0.86)	0.48 (0.45, 0.51)	<0.05	<0.05
RAN	0.80 (0.77, 0.83)	0.57 (0.51, 0.62)	0.83 (0.78, 0.88)	0.46 (0.36, 0.55)	<0.05	<0.05
DenseNet	0.78 (0.75, 0.82)	0.57 (0.52, 0.63)	0.83 (0.81, 0.85)	0.46 (0.39, 0.54)	<0.05	<0.05
ResNext	0.80 (0.76, 0.83)	0.59 (0.54, 0.65)	0.85 (0.83, 0.87)	0.48 (0.47, 0.50)	<0.05	<0.05
Ours	0.85 (0.82, 0.87)	0.70 (0.65, 0.75)	0.89 (0.88, 0.9)	0.62 (0.61, 0.62)		

We calculated P value using Wilcoxon matched-pairs signed-rank test, two-tailed P< 0.05 indicates significant difference. ACC, accuracy; SEN, sensitivity; SPE, specificity; AUC, the area under the receiver operating characteristic curve, P^*^ for AUC; P^#^ for ACC.

**Figure 7:**
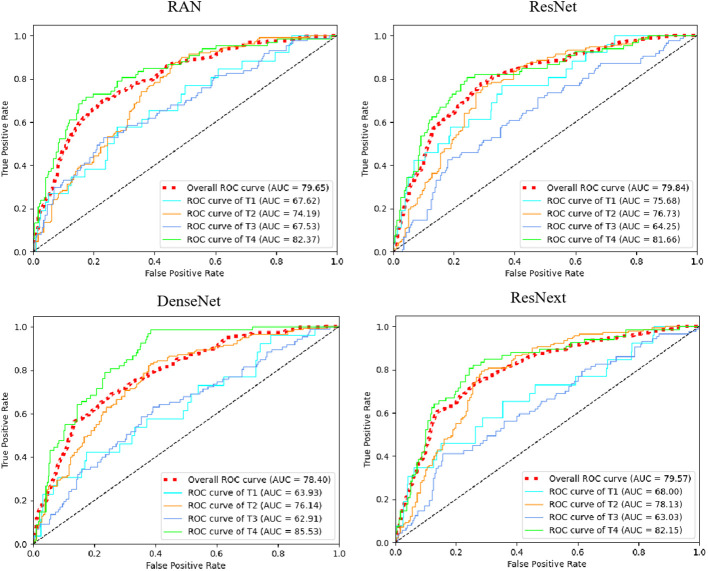
The receiver operating characteristic curves (ROCs) of ResNet, RAN, DenseNet, and ResNext. ROC, receiver operating characteristic; AUC, area under curve. Red dotted line means micro-average AUC, which calculates metrics globally by considering each element of the label indicator as a label.

Additionally, we compared our multi-task model’s predictive performance against other classification models like 3D ResNet-50 (20), RAN (28), DenseNet (29), and ResNext (30). The ROC curves and results are presented in **Figure 7**; **Table 4**. As indicated in **Table 4**, our multi-task model achieved the best AUC, ACC, SPE, and SEN (P< 0.05 for all).

### 3.4 Ablation study

In investigating the contribution of our multi-task design for simultaneously identifying T-stage and segmenting GTV, we have designed two ablation studies: 1) we adopted a 2-step training process (i.e., by first training the segmentation network, then training the T-stage prediction network using the tumor region generated by the segmentation map) and 2) we compared the multi-task model with its degraded version lacking the feature-fusion-aware module (wo-FFA module). In the degraded version, features extracted from segmentation and T-stage prediction tasks were directly concatenated. Employing separate training reduces T-stage prediction performance greatly (see Separate-training in **Table 5**). This reduction may be attributed to the independent nature of segmentation of tumor region, which limits the information transfer between the two related learning tasks, thereby potentially hindering prediction performance. We can observe that the multi-task model trained without feature-fusion-aware module improve the performance compared with the Separate-training in **Table 5**, which indicated the effectiveness of our multi-task design for simultaneously identifying T-stage and segmenting GTV. Upon incorporating the proposed feature-fusion-aware module, our multi-task model achieved best performance (ACC: 0.7 vs 0.61, AUC: 0.85 vs 0.80, SPE: 0.89 vs 0.86, SEN: 0.62 vs 0.57; median DSC: 0.74 vs 0.73, median ASD: 0.97 vs 0.98). This underscores the advantage of the feature-fusion-aware module in refining and fusing features across different tasks and scales.

**Table 5:** Comparison of performance by our multi-task model and its degraded counterparts.

	Classification				Segmentation	
	ACC	AUC	SPE	SEN	DSC	ASD
**Separate-training**	0.54	0.78	0.84	0.53	0.73	1.05
**wo-FFA module**	0.61	0.80	0.86	0.57	0.73	0.98
Ours	0.7	0.85	0.89	0.62	0.74	0.97

DSC, Dice similarity coefficient; ASD, average surface distance; ACC, accuracy; SEN, sensitivity; SPE, specificity; AUC, the area under the receiver operating characteristic curve. Separate-training, a 2-step training process (i.e., by first training the segmentation network, then training the T-stage prediction network using the tumor region generated by the segmentation map); wo-FFA module, lacking the feature-fusion aware module.

### 3.5 Result on the independent dataset

To show the generalization ability of our proposed method, we further conducted experiments on the independent dataset with other 150 subjects. Note that, we used the 150 subjects for testing three proposed multi-task models which were trained on three-fold cross-validation using 320 subjects, and obtained the average result. To quantitatively evaluate classification performance, we reported the results in terms of ACC, AUC, SPE, SEN for T-stage prediction and median DSC, median ASD for segmentation in **Table 6**. As shown in **Table 6**, our proposed method achieved reliable performance, illustrated the good generalization ability of the proposed multi-task model.

**Table 6:** The performance for tumor segmentation and T-staging by our proposed multi-task model in second dataset.

Folds	Classification				Segmentation	
	ACC	AUC	SPE	SEN	DSC	ASD
Fold_1	0.69	0.83	0.87	0.48	0.76	1.23
Fold_2	0.65	0.84	0.85	0.49	0.72	1.68
Fold_3	0.62	0.81	0.86	0.75	0.71	1.50
Avg	0.65	0.83	0.86	0.53	0.73	1.42

DSC, Dice similarity coefficient; ASD, average surface distance; ACC, accuracy; SEN, sensitivity; SPE, specificity; AUC, the area under the receiver operating characteristic curve; Avg, Average.

## 4 Discussion

In this research, we developed a multi-task deep learning model that achieves simultaneous GTV segmentation and T-stage identification in an end-to-end framework. Specifically, we tackle GTV segmentation task with the 3D U-Net architecture and design a transformer-style prediction network for T staging. To integrate the tasks of GTV segmentation and T-stage identification, we introduced a feature-fusion-aware module. We evaluated our proposed model using two sets of CE-T1WI images. The proposed multi-task model has demonstrated remarkable competency in delineating and diagnosing primary GTV in the nasopharynx, achieved comparable contouring results, with a median DSC of 0.74 and ASD of 0.97 mm. It also proved effective in automated T staging, exhibiting an AUC of 0.85 (95% CI: 0.82, 0.87) across 320 patient cases. In comparative studies, our multi-task model outperformed the single-task segmentation network U-Net, achieving better DSC and ASD values (median DSC: 0.74 vs. 0.73; median ASD: 0.97 mm vs. 1.05 mm). Additionally, our multi-task model outperformed single-task classification networks in T-stage prediction, achieving the best results in AUC, ACC, SPE, and SEN. These extensive experimental results underscore that by integrating CNN for GTV segmentation and Transformer for T-staging, our multi-task model leverages the strengths of both architectures, optimizing performance for each specific task, providing a robust and reliable tool for NPC diagnosis and treatment planning.

The Chinese Society of Clinical Oncology (CSCO) 2022 Guidelines for NPC diagnosis and treatment advocate for “stratified administration”, suggesting less intense treatment for patients with lower risk of recurrence or metastasis and more aggressive treatment for those at higher risk (31). Timely diagnosis and personalized treatment plans are crucial for improving the long-term survival of NPC patients. Prior studies have shown that customized target setting for different T-stages can minimize radiation exposure to surrounding structures without increasing recurrence risk (32, 33). Given the scarcity of oncologists/radiologists and the labor-intensive nature of interpreting vast amounts of imaging data, there is a pressing need for more accurate tools to assist with the multitude of follow-up MR scans in NPC patients. To address this need, our objective was to develop a computer aided design (CAD) tool using MR images for simultaneous tumor delineation and T-staging in NPC. Notably, the accuracy of our multi-task model in contouring GTV of NPC on CE-T1WI MR scan was lower than that for detecting primary tumor on multi-sequence MR scans in term of median DSC (0.74 vs 0.79), but achieved higher median ASD (0.97 mm vs 2.0 mm) (3). We hypothesize that integrating data from multiple sequences in future iterations of our multi-task model could further enhance its GTV contouring performance. In previous study focusing on T-stage prediction, a multi-perspective information aggregation framework demonstrated a slightly higher AUC (0.88 vs 0.85) for automatic T-staging in NPC than our multi-task model (34). However, it is important to note that this framework employed a multi-branch architecture with outputs from three parallel branches integrated through major voting, which may have contributed to its enhanced performance. In contrast, our multi-task model was designed as an end-to-end, multi-task network that synchronously outputted tumor region maps and T-stage probabilities, offering a streamlined and efficient approach to NPC diagnosis and staging.

Despite the promising results, our study does have several limitations. Primarily, our reliance solely on CE-T1WI MR scans may have limited the model’s capability by not incorporating the discriminatory power of other MR sequences. To address this, future developments of our multi-task model will involve the integration of data from multiple sequences, aiming to enhance both GTV contouring and T-staging accuracy. Additionally, although our proposed method demonstrated reliable performance on an independent dataset, the generalizability of the model requires further enhancement through studies involving larger and more diverse datasets. Moreover, our model exhibited relatively weaker performance in identifying T1 stage disease compared to other stages. This discrepancy may be attributed in part to the category imbalance among the four T-stages in our dataset. Addressing this imbalance in future iterations could enhance the model’s performance across all stages. Another limitation is that our current model does not fully align with the latest TNM staging system (8th edition), which underscores the importance of considering different combinations of T, N, and M classifications for NPC treatment. Consequently, there is a critical need to expand the model to include nodal gross tumor volume (GTVn) contouring and N-staging, which would offer a more comprehensive approach to NPC staging and treatment planning.

In summary, our study devises a model that can simultaneously identify T-stage and perform accurate segmentation of GTV in NPC. The findings emphasized the potential of multi-task model for simultaneously delineating the tumor contour and identifying T-stage. The multi-task model harnesses the synergy between these interrelated learning tasks, leading to improvements in the performance of both tasks. The impressive performance demonstrates the potential of our work for delineating the tumor contour and identifying T-stage and suggests that it can be a practical tool for supporting clinical precision radiation therapy.

## Data availability statement

The original contributions presented in the study are included in the article/supplementary material. Further inquiries can be directed to the corresponding authors.

## Ethics statement

The studies involving humans were approved by Medical exhics committee of NanFang hospital of Southern Medical University. The studies were conducted in accordance with the local legislation and institutional requirements. Written informed consent for participation was not required from the participants or the participants’ legal guardians/next of kin in accordance with the national legislation and institutional requirements.

## Author contributions

KY: Data curation, Formal analysis, Investigation, Methodology, Validation, Visualization, Writing – original draft. XD: Formal analysis, Investigation, Methodology, Software, Validation, Writing – original draft. FT: Data curation, Formal analysis, Investigation, Visualization, Writing – original draft. FY: Data curation, Investigation, Validation, Writing – original draft. BC: Data curation, Investigation, Validation, Writing – original draft. SL: Conceptualization, Funding acquisition, Resources, Supervision, Writing – review & editing. YZ: Conceptualization, Funding acquisition, Resources, Software, Supervision, Writing – review & editing. YX: Conceptualization, Funding acquisition, Investigation, Methodology, Project administration, Resources, Software, Supervision, Validation, Writing – review & editing.
